# Enhancing time synchronization between host and node stations using a fuzzy proportional-integral approach for EtherCAT-based motion control systems

**DOI:** 10.1371/journal.pone.0324939

**Published:** 2025-06-04

**Authors:** The Tri Bui, Ha Quang Thinh Ngo

**Affiliations:** 1 Faculty of Mechanical Engineering, Ho Chi Minh City University of Technology (HCMUT), Ho Chi Minh, Vietnam; 2 Vietnam National University-Ho Chi Minh City (VNU-HCM), Linh Trung Ward, Thu Duc City, Ho Chi Minh, Vietnam; Sathyambama Institute of Science and Technology: Sathyabama Institute of Science and Technology (Deemed to be University), INDIA

## Abstract

Real-time synchronization for the servo system becomes one of the most popular themes in the Industry 4.0 era. Each connected agent publishes its data and mutual information in the shared protocol while host station plays a role as coordinator among them. In the existing works, it reveals several drawbacks owing to low adaptability and inestimable redundancy. Hence, our study presents a novel method to synchronize between host and slaves by using the modified EtherCAT-based Type-2 Fuzzy-Proportional-Integral (mET2FuPI) control scheme. To serve our purpose, a custom platform of EtherCAT host is investigated to maintain the powerful resource in both data exchange and theoretical computation. The experimental validations of our system including one host station and three node stations are tested to measure the synchronized performance. From these results, it can be obviously seen that our approach is effective and feasible to deploy in the real-world applications.

## 1. Introduction

A real-time control system requires that the tasks are performed at specific or specified points in time. This means that the task execution is time-based mechanism and would be triggered when the system clock reaches the given time. In the centralized control systems, satisfying timing constraint is simple because all the tasks are performed on the central server. But in the distributed control systems, many network nodes are used to undertake a range of specialized tasks. For the general purpose, the nodes have to work very closely together and be synchronized with each other [1].

Time synchronization can be achieved using various wireless and wired techniques of protocols such as Precision Time Protocol (PTP), Network Time Protocol (NTP) or Global Navigation Satellite System (GNSS) [2–4]. Serial buses operating at high speed are widely employed in modern automation networks for integrating the central server with distributed nodes [5,6], especially Ethernet-based network protocols. Accordingly, EtherCAT [7] is among the most significant protocols, widely renowned for its minimum cycle time, topology structure versatility, bandwidth economy, and very accurate time synchronization.

Owing to the Distributed Clock (DC) mechanism, it allows EtherCAT to be used in the distributed real-time control tasks where different nodes or nodes need to coordinate their activities. In this mechanism, it typically selects the first DC node as the reference node, and synchronizes the other DC nodes with the reference node. Since the DC mechanism has superior performance in time synchronization [8,9], delay time between the nodes is approximately 100 ns after measuring the system parameters of this mechanism. Although the synchronization between the host and the nodes is significantly lower, only an initially compensating mechanism between the host and the reference node is needed while there are no measurements of propagation delay, nor any clock drift compensation [10]. Additionally, the jitter time of the host is based on relatively software [11].

Traditional techniques of synchronization regularly have limitations in dynamic environments where factors such as computational load, hardware constraints, and network jitter cause significant errors. Although the proposed controllers perform very well [12,13], they have fixed parameters and lacks of the ability to adapt to altering system conditions, leading to poor performance in environments with noise from various sources. The method of [12] relies on the value of each cycle of each node, which means that if a node fails, the value needed for the next transfer cannot be estimated in advance, leading to a synchronization failure of the entire system. On the other hand, [13] heavily relies on the RTX real-time operating system as well as the averaging method to reduce the impact of inaccurate timestamps, which is insufficient. To overcome these challenges, this investigation proposes a custom low-cost architecture design of EtherCAT host comprising both hardware and programming software as well as exploiting the proposed mET2FuPI scheme to enhance synchronization accuracy in network control systems. To prove the effectiveness and feasibility of our approach, the experimental platform is established to evaluate synchronization performance under the real-world conditions, involving the unexpected noise and varying network loads. From these results, it can be obviously seen that the proposed scheme considerably reduces synchronization errors compared to conventional method [14], and achieves more stable and reliable synchronization process.

The structure of this research is constructed as follows. Section II analyzes the state-of-the-art researches in related works to provide a foundation for our study. Then, section III explains in detail how the DC mechanism operates in the EtherCAT-based system, and identify several factors affecting synchronization accuracy. In section IV, we introduce the embedded solution for EtherCAT host, and depicts its hardware and software architecture. The proposed algorithm mET2FuPI is demonstrated in section V to launch the modified Type-2 Fuzzy-PI structure, compute time compensation, and integrate its into the embedded system. Later, some test scenes are conducted in section VI and analyzed its results. Finally, conclusion and further developments are mentioned in section VII.

## 2. Literature review

There are only three categories in the field of multi-servo control system, as Table 1: distributed clock, clock synchronization, operating system, and communication protocol. Distributed clock mechanisms are widely applied in network-based servo systems for obtaining precise synchronization in various devices. Distributed time synchronization approaches such as EtherCAT are utilized, which offer coordinated actuation and timely coordination between host and node nodes. Methods such as drift compensation and frequency-tracking clock servos (FTCS) improve quality of synchronization, reduce delay, and save bandwidth. For instance, FTCS facilitates rapid acquisition of local clock to a reference, but at the expense of fewer synchronization messages and nanosecond-order accuracy. These systems are generally hardware-config-dependent on configurations, such as clock registers, and real-world implementation-scaled bottlenecks.

**Table 1:** List of the state-of-the-art of review in related fields.

Category(s)	Author(s)	Contribution(s)	Advantage(s)	Disadvantage(s)
Distributed clock	[17] Park et al.	A new synchronization method for distributed systems, including an EtherCAT system consisting of a host, nodes, and an external processor.	Novel: The method only requires the use of general-purpose ports and counter registers on processors to measure and calculate setting time.	Synchronization with the EtherCAT host using an external microprocessor is challenging to implement since, in most cases, an EtherCAT host system only requires one central CPU to conduct all functions.
	[12] Park et al.	A method for improving host-node synchronization accuracy in EtherCAT networks is to implement as part of the host application and does not require any changes to the network protocol, heavy computation load, or huge memory space.	Novel: it does not require changes to the original EtherCAT protocol, burden computational loads or large memory capacity, thus it can be immediately deployed at low cost to the existing EtherCAT networks.	Dependent on the jitter of time of the host system can limit the effectiveness of the method
	[18] Cena et al.	The purpose of this paper is to evaluate several properties of the distributed clock mechanism, specifically its accuracy in achieving coordinated actuation across the network.	Apply: Conducted thorough experiments on actual devices to evaluate the accuracy of the DC mechanism in achieving synchronization effects on the EtherCAT network.	The experiments were only carried out on a small-scale EtherCAT network with at most 12 nodes. Further evaluations on larger networks are needed.
	[19] Park et al.	An innovative method of drift compensation to improve clock synchronization in EtherCAT networks by estimating and compensating for drift of each node at the host, resulting in higher synchronization accuracy.	Improvement: The proposed method significantly improves clock synchronization accuracy in the EtherCAT network, implemented in the current synchronization mechanism without excessively increasing computational load, merely performing simple shifts.	Additional information is needed on how the proposed method is implemented in real-world environments and its scalability. Another characteristic error used to synchronize the reference node with other nodes has not been mentioned.
	[20] Cena et al.	Several aspects of behavior of the distributed clock mechanism are examined, and the accuracy achieved is evaluated using measurements taken on a real network setup.	Apply: The results show that the accuracy achieved is very good, with the average discrepancy between timestamps from different nodes around 10 ns.	The paper only conducted tests on a small-scale network (up to 10 devices), and additional tests on larger networks are required to evaluate feasibility and accuracy under different conditions.
Clock synchronization	[21] Li et al.	A synchronization method for local EtherCAT host-node applications in open CNC systems. They define the initial shift time and calculate it, as well as the clock rate ratio between the host and reference clocks, for the initialization of SYNC0 signal.	Improvement: the calculation of the initial shift time, the clock speed ratio between the host clock and the reference clock, and the remaining cycle time of the host clock in the open CNC system.	The paper mainly focuses on theoretical and simulation aspects without extensive practical testing on a large scale to comprehensively assess the effectiveness of the proposed synchronization method.
	[22] Chen et al.	The FTCS synchronizes the reference clock by modifying the local clock. The frequency of FTCS can be quickly fixed onto the reference frequency, and the offset can be fully compensated within a single synchronizing cycle.	Improvement: FTCS uses fewer synchronization messages, saving real-time resources and bandwidth in networked motion control systems.	The performance of FTCS depends on the controller parameters, which must be precisely tuned to achieve optimal performance.
	[23] Nguyen et al.	An adaptive clock servo algorithm improves time synchronization in network-based control systems. Improving noise reduction results in better synchronization of time offset and rate difference between devices.	Improvement: Introduced a time synchronization method for all network components using a fuzzy-PI algorithm, enabling standalone systems to achieve nanosecond-level or lower clock deviations	Operation depends on clock registers, so systems lacking these registers cannot apply the approach. Employing Fuzzy-PI techniques can increase software system complexity.
	[13] Chen et al.	Unlike traditional synchronization mechanisms, which frequently require a settling time, this method eliminates the settling time, resulting in a faster synchronization between the host and reference clocks.	Novel: This method achieves clock synchronization without requiring processing time and ensures no packet loss even under a general-purpose Windows operating system.	This method shows limitations in achieving high accuracy during synchronization, with lower performance compared to Distributed Clock mechanisms, especially under shorter cycles. It is quite dependent on the RTX real-time operating system, so further research expansion is limited.
Operating system and communication protocol	[24] Ganz et al.	The interaction between PTCP host and EtherCAT node and proposes a new synchronization method for EtherCAT networks.	Improvement: The new synchronization method achieves high quality with minimal jitter, demonstrating feasibility and effectiveness in real-time industrial applications.	Additional techniques are required to integrate PTCP or PTP protocols into EtherCAT and optimize interactions among system components.
	[8] Lee et al.	A method for designing an EtherCAT node module and applying it to a closed-loop motor drive.	Apply: the design and implementation of the EtherCAT Node module, including the use of an ARM Cortex-M3 and ET1100 as the controller. It also specifically describes the actual system deployment, including system configuration, control program development, and motor operation processes.	The paper focuses solely on testing the system with 32 axes in switching closed-loop mode, without clarifying the contributions of study in a broader context.
	[25] Nguyen et al.	The assessment of network latency considers the variations in PLC programming, encapsulated within EtherCAT datagrams, as they traverse Ethernet cables and the EBUS serial medium, being processed and forwarded by modules without DC mechanism support.	Apply: Used TwinCAT PLC software to evaluate network latency and verify the stated hypotheses.	Identified issues related to network latency but did not propose specific solutions to address these problems, which may limit the practical application of the study.
	[26] Cereia et al.	With support from the widely adopted RT Patch real-time extension for Linux, an open-source EtherCAT host undergoes comprehensive long-term testing, reinforcing confidence in its applicability to real-world use cases.	Apply: The paper compares the performance of RT Patch with an RTAI-based system, providing valuable information on the performance and stability of both systems.	The RT Patch system is still affected by certain types of interference load, especially GPU load, which may reduce real-time performance.
	[27] Cena et al.	The study evaluates how network topology and interface types influence accuracy, precision, and real-time characteristics. To achieve this, prolonged measurement campaigns were undertaken, encompassing hundreds of hours of testing on actual network configurations and collecting billions of data points.	Apply: The results show that DC performance on the E-bus is very good, meeting distributed synchronization requirements in motion control applications.	DC performance on Ethernet interfaces is somewhat lower than on the E-bus, and some unwanted behavior (though not severe) occurs, indicating the need for improvements when using Ethernet.

In the second type, high-level algorithms like adaptive clock servo are key in optimizing the stability and synchronization of multi-servo control system. The algorithms dynamically adapt system parameters for the purpose of improving time synchronization among devices to give the system noise robustness and network resistance. Further, the integration of operating systems and communication protocols deeply optimizes the performance of multi-servo systems. The protocols like EtherCAT have been adapted to talk to real-time operating systems like RT Patch or RTX in support of efficient servo module control. There are technologies like the PTCP-EtherCAT synchronization technique that will purportedly mitigate jitter and improve the performance of real-time industrial applications. However, issues do persist relating to the optimization required to compensate for network delay and real-time interference, compromising system performance. Besides that, fuzzy controller design is a common field in intelligent adaptive control systems, meeting the changing conditions and complexity of the controlled objects [15]. Combining the analyses of fuzzy controllers utilized for similar systems [16] with the analyses in the EtherCAT domain is the basis of the proposed solution.

## 3. Principle of EtherCAT DC

In this section, the working principle in the DC mechanism for synchronization is discussed, presenting the synchronization approach between the host and the reference node. First, the clocks and times are determined to use by the host and the node in the DC mechanism. Consequently, system time in the host is assigned by the central clock of the operating system on which the host program runs. Local time in a node is assigned by an internal clock, and it manages the global clock and system time, taking January 1, 2000, as the reference time. The standard format of this reference time which is used to specify the system time of the reference node, is stored in the 64-bit System_Time register with a unit of 1 ns. The reference node is characteristically selected as the node with the DC function located closest to the host. Theoretically speaking, the synchronization process between the host and node consists of three phases: propagation delay measurement, offset compensation, and drift compensation.

### 3.1 Propagation delay

**Fig 1:**
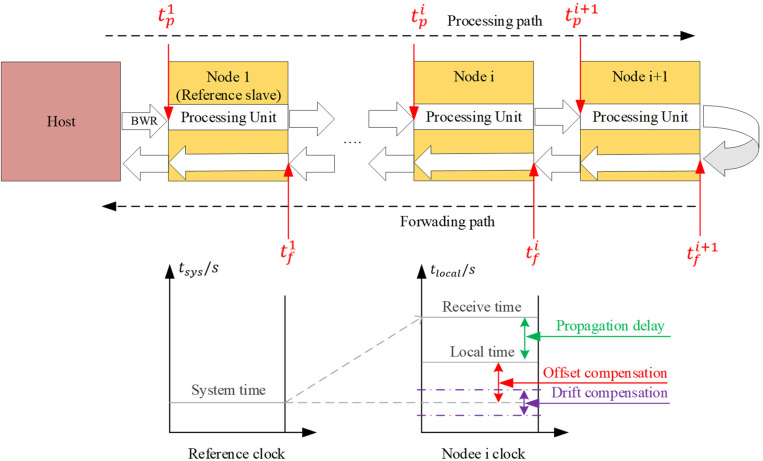
Structure of connected bus for EtherCAT network.

The host initiates the process of propagation delay measurement by sending a broadcast write (BWR) datagram to each node. Whenever a datagram is received at any port of a node, it is recorded a timestamp and stored it in the corresponding Receive_Time_Port registers (0–3). To enlighten more details, a simple network topology is shown in Fig 1 where $t_{p}^{i}$ represents the sequence when the BWR datagram arrives at port 0 of the i-th node in the processing direction, and $t_{f}^{i}$ denotes the sequence when it returns to port 1 of the i-th node in the forwarding direction. In this study, processing direction term involves passing through the EtherCAT processing unit, while forwarding direction term simply goes straight back to the host without going through any additional unit.

When each node completes recording timestamps in the Receive_Time_Port registers, the host collects these timestamps from all nodes. Using this dataset and the known network topology, the host calculates the propagation delay between the reference node and each other node. These computed delays are written to the System_Time_Delay registers of the respective nodes, except for the reference node, where the delay is set to zero, as it serves as the baseline. The propagation delay measurement is executed only once during network initialization, specifically in the Pre-OP state.

### 3.2 Offset compensation

The offset of each node is defined as the difference between the reference time and the local time of each node, which occurs when individual nodes are powered on at different moments. The host reads the local time of each node and calculates its offset by determining the difference between the system time of reference clock and the local time of each node, accounting for propagation delay of each node. Once the offsets are determined, the host writes these values to the System_Time_Offset registers of all nodes in the network. This process is performed only once during network initialization and is essential for rapid delay compensation.

Using the local time and the offset stored in the System_Time_Offset register, each node compensates for its offset by computing the system time as follows:


$$t_{sys}=t_{local}\;+t_{offset}$$ (1)

Where $t_{sys},\;t_{local},\;t_{offset}$ respectively represent the system time, local time, and offset of each node in Fig 1.

### 3.3 Drift compensation

All nodes must synchronize their time with the reference node after compensating for propagation delay and offset. However, over time, the system time of the host and the non-reference nodes drifts from the reference time due to differences in the oscillation frequencies of the network components and jitter in the host clock. To address this, nodes perform drift compensation while in the operation (OP) state. During each cycle or after a specified number of cycles, the host sends an EtherCAT command called “read and multiple write” (FRMW) to distribute the system time of reference clock to the nodes in the network. Each node receives the reference clock time, compares it with its local clock, and adjusts its local clock by calculating a compensation value Δt:


$$\Delta t=t_{local}\;+t_{offset}-t_{prop}-t_{recv}$$ (2)

Where $t_{prop}$ is the propagation delay stored in System_Time_Delay register of each node, and $t_{recv}$ is the time at which the FRMW datagram is received. The resulting value of Δt is stored in the System_Time_Difference register of each node. The clock value is incremented by 10 ns if the average Δt is close to zero, whereas it is incremented by 11 ns or 9 ns when the local time is detected to be slower or faster than the reference time, respectively.

## 4. Conceptual design of target hardware

Host-reference node synchronization is impacted by both jitter and drift in the host clock. Host in an embedded system program is usually subject to events such as interrupts and frame creation latency, thereby introducing increasing synchronization errors in the system. Such errors in high-precision systems may cause data packet loss, consequently impacting motion control significantly. To minimize such errors, two general approaches are taken:

+ Synchronize the host clock with the reference clock: In this technique, the reference clock sends its current time to the host in every cycle, and the host adjusts its clock accordingly. This method is straightforward and takes less resource on the host but may experience deviations because of the lower precision of the host clock. Such deviations can lead to clock drift among the other nodes, which can impact servo trajectories in motion control systems.
+ Adjust the host clock to the reference clock: Since the reference clock on the node side is typically more precise, the host clock is adjusted to it. With this procedure, it is possible to employ various clock adjustment algorithms, again providing greater flexibility to respond to operating conditions when the EtherCAT host is in the OP state. This method is the primary topic of our investigation and is applied to the distributed system synchronization algorithm in EtherCAT.

### 4.1 Design of EtherCAT host

For the generic implementation of an EtherCAT host, synchronization algorithms can be implemented in a flexible manner either using software-based or hardware-based approaches. Software-based implementation is achieved through the configuration of the operating system. Industrial EtherCAT controllers, nevertheless, run on heterogeneous software platforms, which only admit restricted adaptability since manufacturers do not release source code. This calls for the requirement to develop various versions for every platform. In addition, a change in the operating system reduces software flexibility and complicates updates because protocol stack changes can clash with new functionality that is introduced by system updates. Another alternative is to employ a higher-performance Central Processing Unit (CPU), but this increases the microprocessor cost. High-performance processors are also less desirable for embedded controllers, which must run continuously over extended periods of time, due to increased heat dissipation, power consumption, and cooling requirements—parameters that increase failure rates and upkeep. To alleviate these issues, our work discusses custom hardware development of main EtherCAT processing. The design supports frame configuration and sequential cyclic execution while communicating in parallel with a PC. It offers high communication rates like traditional EtherCAT controllers but with reduced dependency on PC platforms.

**Fig 2:**
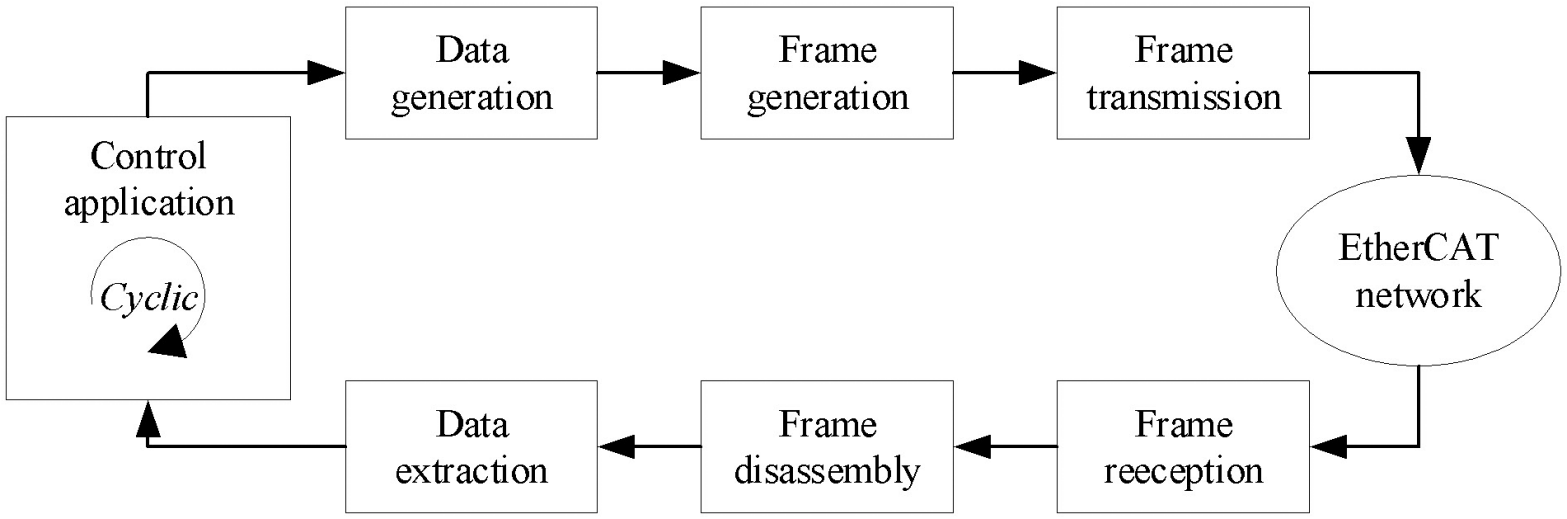
Communicative procedure in the proposed EtherCAT host.

The design proposed in this paper supports frame configuration and cyclic execution through dedicated hardware specifically developed for primary EtherCAT processing while maintaining parallel communication with a PC. The hardware design aims to achieve high communication speeds comparable to traditional EtherCAT controllers while reducing dependence on PC platforms. To achieve high-speed communication cycles, the proposed host firmware manages frame creation, segmentation, transmission, reception, and execution, as illustrated in Fig 2. This host architecture is compatible with various platforms while maintaining highly accurate cyclic communication.

The steps for controlling, configuring the EtherCAT system, and extracting data are highly dependent on user applications and must be executed on the software of PC. The interface between the EtherCAT host and the PC needs to be carefully designed for these operations. To minimize data transmission processing time and simplify software development, the communication protocol must be compatible with all operating system platforms and have pre-existing drivers available without requiring additional installation.

### 4.2 Main hardware platform

**Fig 3:**
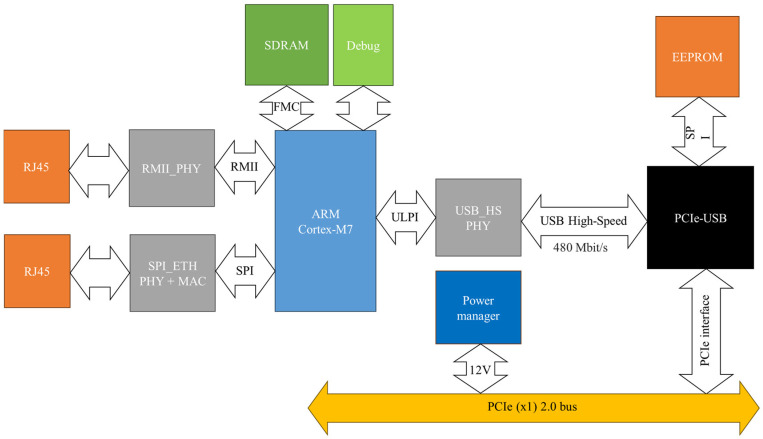
Hardware platform of the proposed EtherCAT host.

The hardware design of EtherCAT host is critical, as it must incorporate all essential functions of an EtherCAT host in compliance with specified standards, ensuring that outgoing datagrams meet strict timing requirements. Specifically, the maximum communication cycle between the EtherCAT host and nodes is set at 1 ms to maintain high servo control accuracy. The primary CPU of embedded host module can be a microprocessor or microcontroller (MCU) but must operate at a sufficiently high frequency to handle all tasks before transmitting and receiving datagrams. However, higher operating frequencies increase energy consumption and costs. To address this, an MCU-based EtherCAT host hardware architecture is presented in Fig 3. This simplified EtherCAT controller meets the functional and performance requirements of more complex controllers, with the advantage of being easy to use and not requiring specialized hardware. All that is needed is a PC with a PCI Express (PCIe) slot running any operating system with USB drivers.

The key components of the proposed PCIe EtherCAT host include an STM32H7 MCU as the CPU, Ethernet PHY ICs, a PCIe–USB interface, storage components, and a power management circuit to convert a 12 V input to the required voltages. The CPU, based on a high-performance 32-bit ARM Cortex-M7 RISC core, operates at up to 480 MHz but is configured to run at 400 MHz. The EtherCAT host cycle is generated by a 200 MHz timer interrupt module, allowing synchronization cycle adjustments in 5 ns increments. Additionally, the CPU features efficient instructions, a data cache, and 128 KB of tightly coupled memory (DTCM) for real-time data storage, enhancing computation speeds. With a hardware floating-point unit and a DSP instruction set capable of single-cycle execution, the MCU offers low power consumption of approximately 275 µ/MHz during active operation and only 2.43 µA in standby mode.

**Fig 4:**
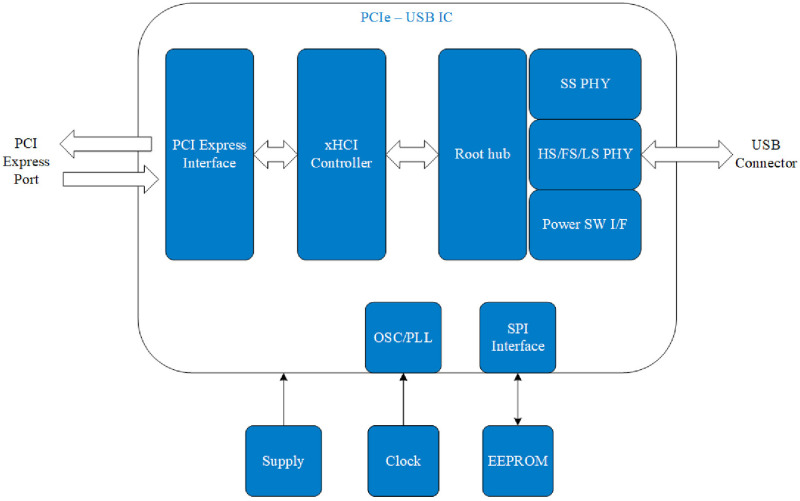
Function block diagram of PCIe-USB interface.

To communicate with a PC, widely available ports on current and older mainboards are utilized. The proposed design uses the PCIe port, as traditional PCI slots are being phased out in modern mainboards, making a PCI-based EtherCAT host less viable. Since most MCUs do not include integrated PCIe protocol handling due to their relatively low operating frequency, USB High-Speed communication is chosen for user interaction. A PCIe–USB interface IC is employed as the USB host controller. Fig 4 illustrates the structure of the PCIe–USB IC, which integrates a single-lane PCI Express Gen2 interface supporting both the link and PHY layers. Functional blocks include:

+ SS PHY and HS/FS/LS PHY: Physical layers for SuperSpeed and Hi-/Full-/Low-Speed data transmission.
+ Power SW I/F: Controls port power and detects overcurrent conditions.
+ Root hub: Operates as an internal hub for the host controller.
+ SPI interface: Connects to external EEPROM memory, enabling firmware updates.
+ xHCI Controller: Manages USB operations, data exchange with peripherals, and operating system communication.
+ PCI Express Interface: Consist of link and PHY layers for communication via PCIe.

### 4.3 Cable redundancy hardware design

The CPU includes peripherals that support both Media Independent Interface (MII) and Reduced Media Independent Interface (RMII) communication standards [28], enabling interaction with a standard 100M Ethernet PHY. EtherCAT itself relies on standard Ethernet hardware for bus communication. Consequently, designing an EtherCAT network interface does not heavily depend on specialized PHY ICs, provided they are compatible with the CPU in use. Additionally, a second network port, referred to as the redundancy bus, is incorporated to compensate for communication cable failures within the EtherCAT system. Both cyclical and non-cyclical data frames are transmitted simultaneously through both ports, traversing the system. Under normal conditions, the primary port ensures that all EtherCAT nodes are accessed and processed in the forward direction, while the secondary port ensures all nodes are also reached in the reverse direction, leaving data in the redundancy frames unchanged.

**Fig 5:**
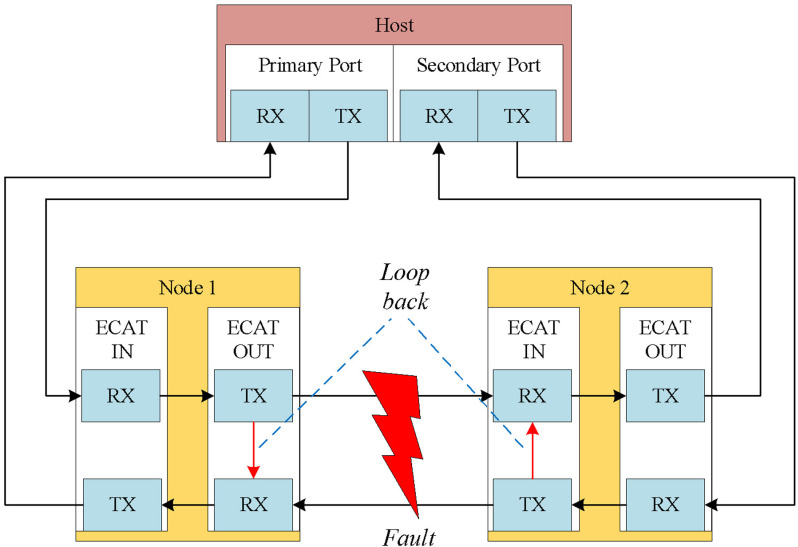
Description of the EtherCAT communication redundancy.

Upon receiving a data frame, the EtherCAT controller verifies the integrity of the input data, which may have been altered during transmission. In the event of a cable break, both data frames are processed independently on either side of the break, with each frame containing a portion of the input data, as shown in Fig 5. The controller then merges the two frames into a single frame and verifies its validity by summing the activity counters from each frame. Whether an EtherCAT node is accessed via the primary or redundant port does not impact processing. The controller ensures data consistency by using appropriate identifiers or mechanisms to match frames, even when data frame of one side is lost during transmission.

The cable redundancy of EtherCAT can tolerate a single fault, ensuring uninterrupted communication with nodes when a cable break occurs. Once the connection is restored, the original communication path is reestablished. However, if multiple failures occur, communication will be interrupted, and all connections must be reinitialized before further operations can resume. Since the CPU used supports only one Ethernet protocol, adding an additional port requires an intermediary SPI–Ethernet interface IC capable of generating custom frames. The EtherCAT bus communication on the secondary port must utilize a DMA channel linked to the SPI peripheral of CPU, with the communication frequency configured according to the performance of IC.

### 4.4 Firmware MCU design

**Fig 6:**
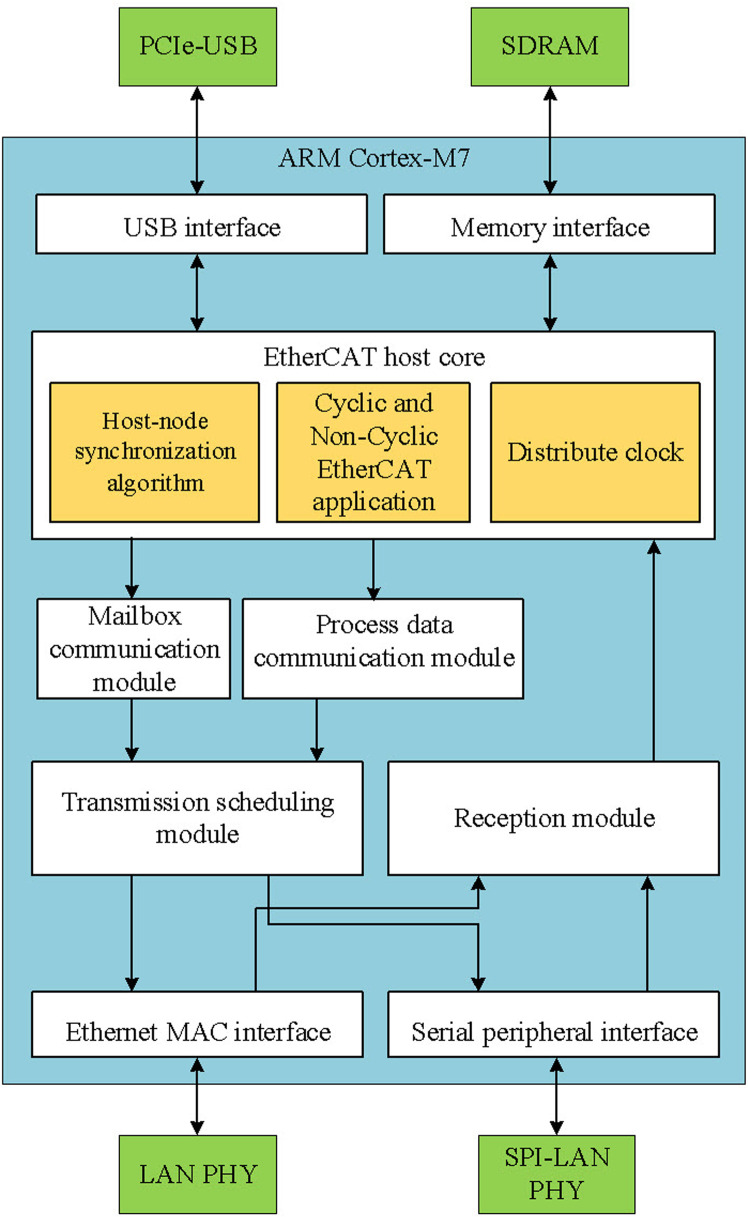
Our architecture of firmware.

The hardware system described above requires a lightweight EtherCAT host software system that can be embedded on an MCU without requiring an operating system, particularly for the ARM architecture. It must also allow algorithm customization. Fig 6 presents the software system structure discussed in this paper, which is built using the library and defined registers of CPU manufacturer [29,30].

EtherCAT communication is divided into two categories: process data communication and mailbox communication. Process data communication is used for transmitting real-time data on a fixed cycle, which can only be modified upon exiting the OP state. This type of communication also supports command datagrams for synchronizing the clocks of devices in the system, although the number of frame formats and types is more limited compared to mailbox communication. Conversely, mailbox communication handles non-real-time data transmission and can be sent to EtherCAT nodes at any time. Applications of mailbox communication include configuration protocols such as CANOpen over EtherCAT (CoE) and Ethernet over EtherCAT (EoE), resulting in a broader variety of frame formats and types than those in process data communication.

The EtherCAT host core block plays a central role, containing the application, synchronization algorithms, and overall clock management for the EtherCAT host. This block sends commands to either the mailbox communication module or the process data communication module based on the communication purpose and receives information from the reception module. The mailbox communication module serves as a buffer for mailbox communications, discarding data that cannot be framed by the transmission module in time. Meanwhile, the process data communication buffer is packaged cyclically by the transmission module, granting it higher priority to prevent significant errors when transmitting motion control commands. The reception module separates incoming frames, distinguishes between process data and mailbox communications, classifies these two communication types, and assigns identifiers before forwarding them to the buffer inside the EtherCAT host core.

**Fig 7:**
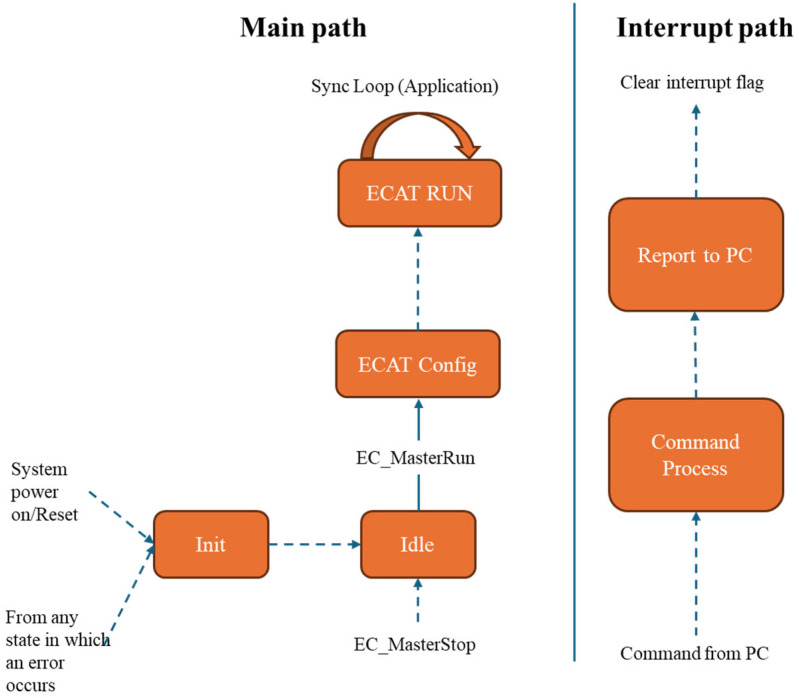
State diagram of EtherCAT host.

In Fig 7, the operation of system begins when power is supplied to the entire system. The MCU initializes, checks all interfaces, and verifies the functionality of the corresponding ICs. If any protocol other than USB fails, the system logs the error and resets the interfaces. The MCU then enters Standby mode, continuously sending status messages to the PC via USB. In the event of a USB communication error, the debug port is used for troubleshooting.

After startup, the EtherCAT host core executes an initial procedure to scan the network, check for connected devices, and store EtherCAT node information in SDRAM. It then waits for commands from the PC to move into the run state and automatically adjusts datagram length and format according to the information saved in SDRAM. If no nodes are connected, the EtherCAT host remains in a waiting state until it receives another command to perform a scan.

## 5. The proposed algorithm for host-node synchronization

**Fig 8:**
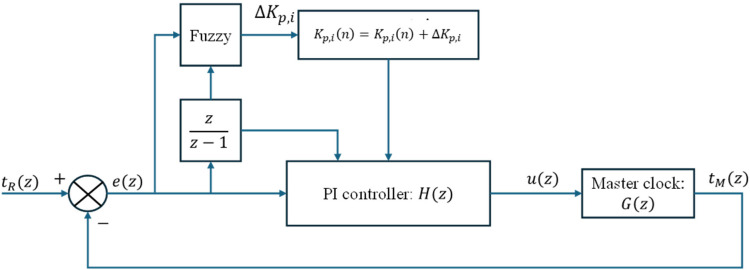
Block diagram of the proposed scheme for host-node synchronization.

A high-precision synchronization system, such as one used for motion control, can be created based on the EtherCAT protocol and an adaptive synchronization algorithm in various scenarios. The research objective of this paper is to design an algorithm that offers fast convergence and high synchronization accuracy, suitable for implementation on microcontroller-based embedded systems running at moderate clock frequencies. Fig 8 shows the clock model and the Type-2 Fuzzy PI adaptive synchronization controller for the host–nodes. This controller can automatically adjust the compensation value when the error changes during both the startup state and the communication cycle.

### 5.1 Clock model

Because the algorithm will be applied in an embedded system, the clock model is considered in the discrete domain, the host clock model as in equation (3). If we assume the cycle is a constant $T_{cycle}$ and it only changes after each system restart, we have the ideal clock equation (4).


$$t_{M}(n)=t_{M}(n-1)+u(n-1)\left[t(n)-t(n-1)\right]$$ (3)

$$t_{M}(n)=t_{M}(n-1)+u(n-1)T_{cycle}$$ (4)

Where $t_{M}(n)$ is the time of host value at the n-th cycle, u(n) is the control variable. By discretizing equation (4) in the z-domain:


$$G(z)=\frac{t_{M}(z)}{u(z)}=\frac{T_{cycle}}{z-1}$$ (5)

### 5.2 The structure of type-2 Fuzzy system

**Fig 9:**
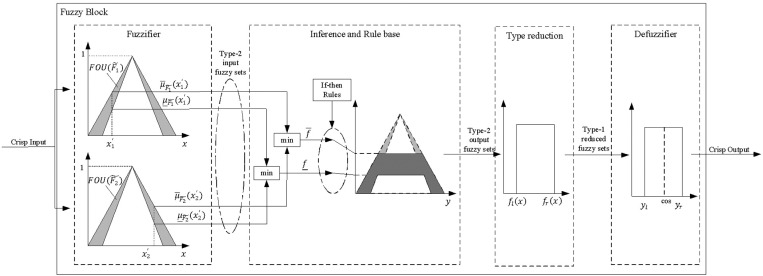
Block diagram of the proposed type-2 fuzzy for each rule.

Because Type-1 fuzzy systems use crisp membership function values that make it difficult to fully capture data uncertainty, a Type-2 fuzzy inference system can provide better output predictions, especially in distributed systems affected by environmental factors. Therefore, this section presents the model and computation methods for the fuzzy block primarily used in Fig 8, in which the four components of the Type-2 fuzzy inference system are detailed in Fig 9.

The details of the type-2 fuzzy components are described as follows:

+ Fuzzifier: With Type-2, the fuzzifier switches the crisp input vector with n elements $x={\left(x_{1},\ldots ,x_{n}\right)}^{T}\in X_{1}*X_{2}*\ldots *X_{n}$ into Type-2 input fuzzy sets $F_{x}$. In Interval Type-2 form, fuzzification results for each input value $x_{n}$ is an interval of membership rather than a single number as in Type-1. Where $\underset{―}{\mu_{F_{x}}}(x),\overset{―}{\mu_{F_{x}}}(x)$ are respectively the lower and upper membership functions of a Type-2 fuzzy singleton fuzzifier [28].
+ Inference and Rule base: The fuzzy system relies on “If-then” rules to map inputs to outputs. For each fuzzy rule i∈(1,2,…,M) among M fuzzy rules, a typical rule takes the form:

$$R^{i}:IF\;(x_{1}\in F_{1}^{i}\;AND\ldots AND\;x_{n}\in F_{n}^{i})\;THEN\;(y_{1}\in G_{1}^{i}\;AND\ldots AND\;y_{c}\in G_{c}^{i})$$ (6)

Where $F_{j}^{i}$ is the antecedent fuzzy set with $x_{j}\;(j=1,2,\ldots ,n)$, $G_{k}^{i}$ is the consequent fuzzy set with $y_{k}\;(k=1,2,\ldots ,c)$. The inference process for each rule is shown in Fig 9, and each rule has a firing level represented as an interval rather than a single value. The upper and lower firing levels $\overset{―}{f},\underset{―}{f}$ are computed as:


$$\overset{―}{f}=min[\overset{―}{\mu_{F_{1}}}\left(x_{1}\right),\overset{―}{\mu_{F_{2}}}\left(x_{2}\right)]$$ (7)

$$\underset{―}{f}=min[\underset{―}{\mu_{F_{1}}}\left(x_{1}\right),\underset{―}{\mu_{F_{2}}}\left(x_{2}\right)]$$ (8)

+ Type-reducer: The inference result from the previous step is still a Type-2 fuzzy set. This step reduces the set from Type-2 to Type-1. Various methods exist for type-reduction. In this paper, an approach similar to the center-of-sets algorithm [29] is used to find the centroid of the Type-2 fuzzy set. The Type-1 reduced fuzzy set for each rule can be expressed as:


$$Y_{\cos}(x)=[y_{l},y_{r}]$$ (9)

Where:


$$y_{l}=\min_{l\in [1,M-1]}\frac{\sum_{k=1}^{l}{\overset{―}{f}}_{k}\;\underset{―}{y_{k}}+\sum_{k=l+1}^{M}{\underset{―}{f}}_{k}\;\underset{―}{y_{k}}}{\sum_{k=1}^{l}{\overset{―}{f}}_{k}\;+\sum_{k=l+1}^{M}{\underset{―}{f}}_{k}\;}$$ (10)

$$y_{r}=\max_{r\in [1,M-1]}\frac{\sum_{k=1}^{r}{\underset{―}{f}}_{k}\;{\overset{―}{y}}_{k}+\sum_{k=r+1}^{M}{\overset{―}{f}}_{k}\;{\overset{―}{y}}_{k}}{\sum_{k=1}^{r}\;{\underset{―}{f}}_{k}+\sum_{k=r+1}^{M}{\overset{―}{f}}_{k}}$$ (11)

In these expressions, L and R are switching points determined by ${\underset{―}{y}}_{L}\leq y_{l}\leq {\underset{―}{y}}_{L+1},\;{\overset{―}{y}}_{R}\leq y_{r}\leq {\overset{―}{y}}_{R+1}$, ${\left\{{\overset{―}{y}}_{k}\right\}}_{k=1}^{M}\;and{\left\{{\underset{―}{y}}_{k}\right\}}_{k=1}^{M}$ sorted in ascending order separately.

+ Defuzzifier: Finally, after the type-reduction step, each rule produces an interval set $Y_{\cos}$ for type-reducer. The defuzzification methods we use are suitable for real-time processing and are implemented in the low-cost embedded system[30]. In this last block, the crisp output Y is calculated by taking the average of the left and right endpoints $y_{l},y_{r}$:


$$Y=\frac{y_{l}+y_{r}}{2}$$ (12)

Thus, this section describes the steps and formulas for the fuzzy block used. The setup parameters, as well as the specific membership functions and rules for the compensation algorithm, will be presented in the following section.

### 5.3 Type-2 Fuzzy PI compensation controller

EtherCAT can compensate for the time discrepancy among nodes through the DC mechanism. However, it is not sufficiently stable for use in micro-scale or synchronous motion systems if the EtherCAT host fails to reliably handle outgoing data packets, such as in robotic or high-precision mechanical applications. A Type-2 Fuzzy PI compensation controller is implemented to achieve high-level compensation performance on the order of nanoseconds. PI compensation is used to correct clock offset and drift speed. Although filters may reduce errors, the parameters $k_{P}$ và $k_{I}$ are fixed during the design process, which may not provide optimal performance if time synchronization requires high frequency or if the system experiences many interrupts. A method is needed with a short response time and adaptable PI compensation parameters. Therefore, using fuzzy, especially Type-2 fuzzy for systems with delay components, enables the host clock to attain a short settling time and ensure time synchronization without requiring training time to update weights. Typically, PI compensation can be described in the discrete domain as follows:


$$H(z)=\frac{u(z)}{e(z)}=k_{P}+k_{i}\frac{z}{z-1}$$ (13)

Combining equations (5), (11) together, the closed-loop transfer function equation is described as follows:


$$D(z)=\frac{t_{M}(z)}{t_{R}(z)}=\frac{G(z)H(z)}{1+G(z)H(z)}=\frac{T_{cycle}(k_{p}z+k_{i}z-k_{p})}{z^{2}+\left(k_{p}T_{cycle}+k_{i}T_{cycle}-2\right)z+\left(1-k_{p}T_{cycle}\right)}$$ (14)

Set $K_{P}=T_{cycle}k_{p},\;K_{i}=T_{cycle}k_{i}$, transmission function of D(z) is rewritten as:


$$D(z)=\frac{(K_{p}+K_{i})z-K_{p}}{z^{2}+\left(K_{p}+K_{i}-2\right)z+\left(1-K_{p}\right)}$$ (15)

Considering the characteristic equation of the denominator D(z)


$$z^{2}+\left(K_{p}+K_{i}-2\right)z+\left(1-K_{p}\right)=0$$ (16)

Roots $z_{1},\;z_{2}$ of this equation is determined by the formula:


$$z_{1,2}=\frac{-\left(K_{p}+K_{i}-2\right)\pm \sqrt{{\left(K_{p}+K_{i}-2\right)}^{2}-4(1-K_{p})}}{2}=\frac{-\left(K_{p}+K_{i}-2\right)\pm \sqrt{{\left(K_{p}+K_{i}\right)}^{2}-4K_{i}}}{2}$$ (17)

For the system to be stable, both roots must satisfy $\left|z_{1}\right|,\left|z_{2}\right|<1$ [31] and $K_{P},\;K_{i}$ must be real numbers. Thus, the stability condition of D(z) is:


$$\begin{array}{c} \left\{ \;\;\;\begin{array}{c} 0\leq K_{p}\leq 1\\ 0\leq K_{i}\leq 4\\ {\left(K_{p}+K_{i}\right)}^{2}>{4K}_{i} \end{array}\right.\; \end{array}$$ (18)

**Fig 10:**
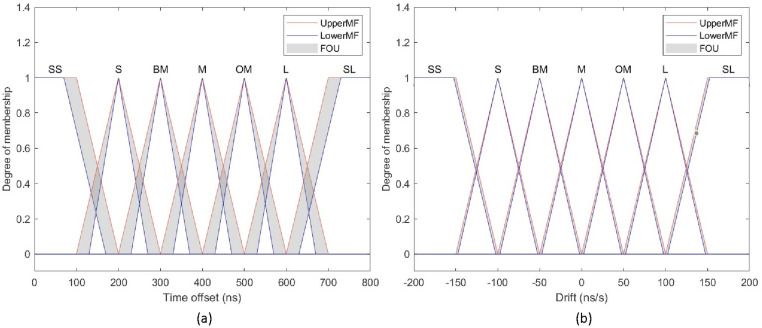
Fuzzy input (a) Time offset (b) Drift.

**Fig 11:**
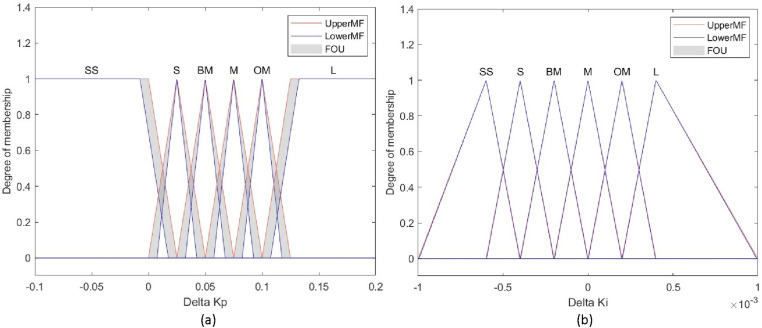
Fuzzy output (a) Delta P control (b) Delta I control.

The specific proof process of the equation (18) is explained in [32]. In the initial phase, the system requires a large time offset value to reach the steady-state value quickly, and the compensation includes more random noise. Once the system is stable, an appropriate drift value is needed to eliminate noise. In this paper, the time offset e and the derivative of the offset de are the two inputs of the fuzzy controller block. The membership functions of these inputs are shown in Fig 9. To smooth the clock time being controlled, the coefficient $K_{p,i}$ is incremented by a delta $\Delta K_{p,i}$ from the fuzzy output (10). The membership functions of the input, output are shown in Fig 10, Fig 11, along with the rule tables in Table 2 and Table 3.

**Table 2:** Fuzzy rule Delta P control.

e/de	SS	S	BM	M	OM	L	SL
**SS**	S	S	BM	M	M	OM	L
**S**	S	BM	BM	M	OM	OM	L
**BM**	BM	BM	BM	M	OM	OM	OM
**M**	M	M	M	M	OM	L	OM
**OM**	M	OM	OM	OM	L	L	L
**L**	L	L	L	OM	L	L	L
**SL**	L	L	OM	OM	L	L	L

**Table 3:** Fuzzy rule Delta I control.

e/de	SS	S	BM	M	OM	L	SL
**SS**	L	L	OM	OM	M	M	M
**S**	L	OM	OM	M	M	BM	BM
**BM**	OM	OM	M	M	BM	S	S
**M**	OM	M	M	BM	S	S	SS
**OM**	M	M	BM	S	S	SS	SS
**L**	M	BM	S	S	SS	SS	SS
**SL**	M	BM	S	SS	SS	SS	SS

The fuzzy logic block adjusts the PI controller values based on human understanding of clock synchronization. Seven fuzzy sets are designed for the time offset and drift: super small (SS), small (S), below medium (BM), medium (M), over medium (OM), large (L), and super large (SL). The delta $\Delta K_{p,i}$ contains six fuzzy sets: SS, S, BM, M, OM and L. In this paper, the minimum value, maximum value, and lower lag value for the two fuzzy inputs are respectively set to 0 ns, 800 ns, and 0.3 for the time offset, and −200 ns, 200 ns, and 0.05 for the drift. The fuzzy output depends on experience gained from the performance tests of system. Therefore, $\Delta K_{p}$ angles from −0.1 to 0.2, with a lower lag value of 0.03, while $\Delta K_{i}$ ranges from −0.001 to 0.001, with a lower lag value of 0.005.

The input and output values are chosen based on the algebraic ranges of the respective sets. The principle behind the fuzzy rules is as follows:

+ Initial phase: When e and de are large or super large, the gain of $K_{p}$ increases rapidly with a maximum value of 0.2 in each cycle to reduce the rise time of the host clock compared to the reference clock, allowing the error to quickly approach the desired value. Meanwhile, the gain of $K_{i}$ needs to be reduced to mitigate the overshoot caused by $\;K_{p}$ and not affect the rise time. Thus, it only takes about 3–4 cycles for e and de to reach the medium value. In this situation, the main sources of error are random noise and quantization errors.
+ Moderate phase: If e and de are of moderate magnitude, but not small enough to transition to a stable state. The gain of $K_{p},\;K_{i}$ set at a moderate level. This allows the controller to continue reducing errors caused by noise or interrupts, but not too much to avoid causing oscillations or overshoot. Next, the error e continues to decrease steadily from the average level towards a smaller level.
+ Steady-state phase: If e and de are small, the clock shift is kept sufficiently small, maintaining an adaptive noise-reduction capability. At this moment, only de value changes rapidly. Although this value changes continuously, it will be quickly compensated within one network cycle.

For the initial controller coefficients, $K_{p}$ should be set to a large value and $K_{i}$ n to a smaller value because, at the first sample, the time offset is very large, necessitating rapid convergence primarily achieved.

## 6. Experimental results

### 6.1 System setup

**Fig 12:**
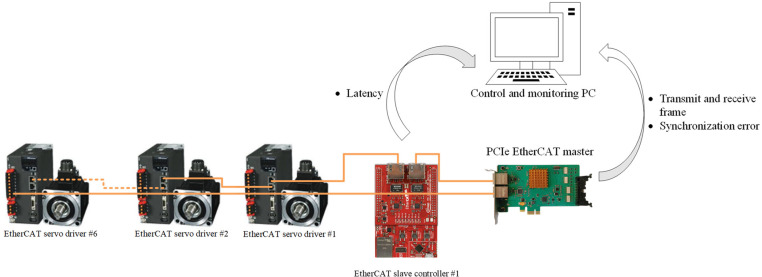
Illustration of our experimental setup.

Due to our design, several experiments are conducted to assess the effectiveness and feasibility of synchronization performance in the proposed system. In Fig 12, host PC issues the control command and gathers data to analyze from the PCIe EtherCAT host which features a PCIe x1 port and runs on a Windows operating system with a built-in USB driver. For the EtherCAT node controller (ESC), Infineon XMC4800 plays a role as the reference, and RS-Automation CSD7 is chosen for the EtherCAT driver—both are commercially available products. The programmable ESC is deployed to send data of time delay to host for analysis.

### 6.2 Performance of PCIe EtherCAT host

The real-time performance of the EtherCAT host system is crucial, as it must complete its tasks within a defined timeframe. To evaluate this, we measured the execution times of two main components of the system: latency, and frame reception/sending time. The latency represents real-time performance by comparing the expected execution time to the actual execution time for each cycle. Therefore, latency is calculated as $t_{latency}=t_{real}-t_{desired}$, where $t_{real}$ is the actual start time of the next communication, and $t_{desired}$ is the intended communication time without the algorithm. The send/receive component covers the period from when the EtherCAT host creates the frame until it fully receives and decodes the return frame from the EtherCAT node.

**Fig 13:**
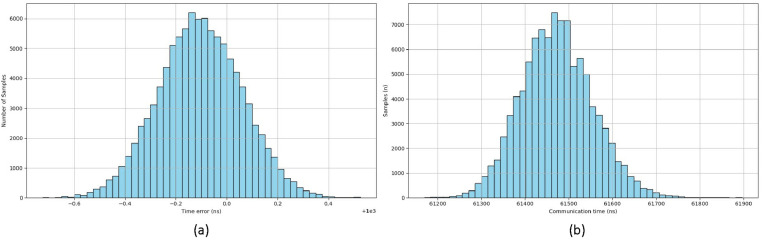
Execution time histogram (a) Latency (b) Transmit and receive.

The results of the 1 ms test case over 100,000 samples, including average deviation, as well as minimum, maximum, and mean values for latency and send/receive time, are presented in Table 4 and illustrated in Fig 13. The findings demonstrate that the EtherCAT system implemented here can adequately perform the required functions within the allowable time for a 1 ms cycle, ensuring sufficient real-time capability for other operations.

**Table 4:** Execution time comparison at 1ms communication (ns).

Execution	AVG	MIN	MAX
**Latency**	−108.4	−725	525
**Send and Recv**	61472.66	61170	61900

### 6.3 Performance of synchronization algorithm

**Fig 14:**
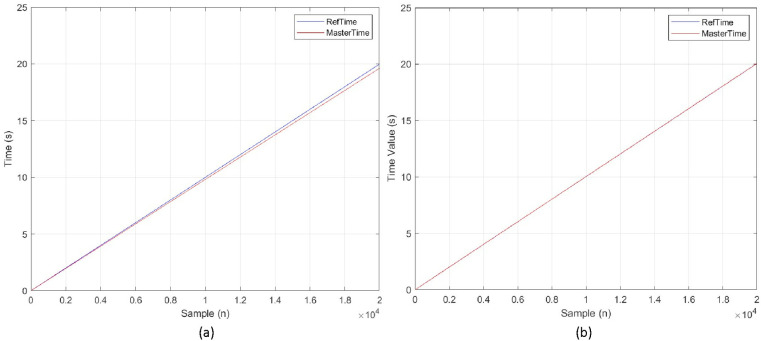
Performance of Host time and reference time with (a) PI controller (b) mET2FuPI controller.

We conducted several tests to evaluate the proposed method based on the EtherCAT protocol using two different controllers, namely the Type-2 Fuzzy-PI controller and a regular PI controller. In this paper, the initial controller coefficients were set to $K_{p}=0.5$ và $K_{i}=0.02$; these same values were used in the PI controller to compare the performance of the proposed method. Interrupt noise and other peripheral interferences were also introduced to assess the algorithm, with timer noise generated every cycle of the EtherCAT bus with lag levels from ±100 ns to ±10,000 ns. The results in Fig 14 show the times of the EtherCAT host and the reference node, where in the first 20 samples, the time of host gradually deviates from that of the reference node. Since the experimental cycle is 1 ms, this corresponds to a total time of 20 seconds. Under the PI synchronization controller, the clock of host deviates by as much as 0.372 seconds from the reference—an extremely large disparity for a real-time system. In contrast, with the proposed controller, this deviation is compensated under all noise scenarios, and the time of host closely matches the reference time.

**Fig 15:**
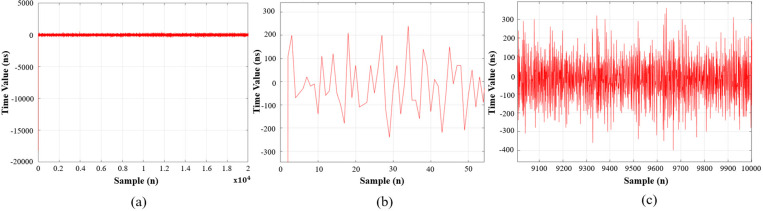
Experimental result of the synchronized error with PI controller, (a) $2\times 10^{4}$2×104 samples, (b) from initial stage to 50 samples and (c) from 9100 samples to 10000 samples.

**Fig 16:**
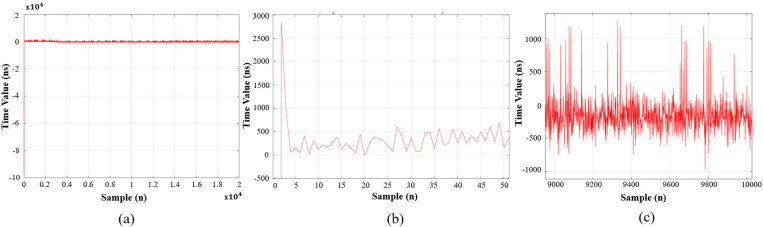
Experimental result of the synchronized error with mET2FuPI controller, (a) $2\times 10^{4}$2×104 samples, (b) from initial stage to 50 samples and (c) from 9100 samples to 10000 samples.

Fig 15a and Fig 16a show the clock synchronization error results. Both experiments have different offset time but reach the steady-state value within the first 50 samples. In Fig 15c, the clock synchronization error only in the range of approximately −400 ns to 400 ns, whereas the clock shift in Fig 16c ranges from about −1000 ns to 1100 ns, demonstrating that the mET2FuPI adapts better to noise types, and can compensate immediately at any value to achieve time synchronization for the entire EtherCAT network.

Table 5 presents the synchronization errors for both algorithms. Lower synchronization errors correspond to a more stable gap between the sync0 signal and the next cycle. Maintaining a stable gap from the transmission time of host to sync0 helps avoid packet loss more effectively. Consequently, the proposed method significantly mitigates packet loss issues.

**Table 5:** Synchronization error in different controllers (ns).

	AVG	MIN	MAX	STD
mET2FuPI	−22.76	− 880	520	177.53
PI	−90.2	−18120	2835	760.9

In Table 6, a comparative experiment was established to evaluate the synchronization performance of mET2FuPI. Park et al. [12] and Libo et al. [10] proposed a synchronization method between EtherCAT host and nodes, host and reference node to node (HNTN) and adaptive synchronization algorithm (ASA) respectively. By modifying the system time of each node rather than the host system time, MSTS was able to achieve host–node synchronization. Both ASA and mET2FuPI were demonstrated in an embedded system based on STM32H7 with a frequency of 400MHz. Therefore, the maximum accuracy of the interrupt module in the host timer is 50 ns, while HNTN, based on embedded Linux, achieves an accuracy of up to 5 ns. The results in the table show the effectiveness of mET2FuPI when compared to published algorithms. Among them, the average magnitude of synchronization error of mET2FuPI is 22.76 ns compared to 42.296 ns of ASA with the same accuracy of 5 ns. In addition, the magnitude of synchronization error of HNTN is better by 5.092 ns compared to mET2FuPI due to the frequency limitation of the interrupt module.

**Table 6:** Synchronization error in different methods (ns).

	AVG	MIN	MAX	Accuracy of interrupt module
mET2FuPI	−22.76	− 880	520	50
ASA	42.296	−749	651	50
HNTN	17.668	−50	104	5

## 7. Conclusion

In this study, a novel concept to synchronize between host and node stations using the proposed mET2FuPI scheme for EtherCAT-based motion control system was presented. The proposed hardware platform provided the high-speed exchange data, powerful computation, and reliable communication to interconnect among stations. Owing to the EtherCAT protocol, rapid transmission of message and scalability for more connected stations are guaranteed. Our mET2FuPI scheme was developed to integrated in the firmware level for adaptive synchronization.

Further development should be indicated. Advanced learning methods such as reinforcement learning strategy could be deployed to improve the behavior of EtherCAT-based motion control system even if some unexpected factors or uncertainties exist. Also, extendable integration with the vision-based approach might be discussed to interact with the working environment.

## Supporting information

S1 File Related data for this research. (RAR)
